# Citation Classics in Systematic Reviews and Meta-Analyses: Who Wrote the Top 100 Most Cited Articles?

**DOI:** 10.1371/journal.pone.0078517

**Published:** 2013-10-14

**Authors:** Olalekan A. Uthman, Charles I. Okwundu, Charles S. Wiysonge, Taryn Young, Aileen Clarke

**Affiliations:** 1 Warwick Centre for Applied Health Research and Delivery (WCAHRD), Division of Health Sciences, Warwick Medical School, the University of Warwick, Coventry, United Kingdom; 2 International Health Group, Liverpool School of Tropical Medicine, Liverpool, United Kingdom; 3 Centre for Evidence-Based Health Care, Faculty of Medicine and Health Sciences, Stellenbosch University, Tygerberg, South Africa; 4 South African Cochrane Centre, South African Medical Research Council, Tygerberg, South Africa; 5 Division of Health Sciences, Warwick Medical School, the University of Warwick, Coventry, United Kingdom; State University of New York, Oswego, United States of America

## Abstract

**Background:**

Systematic reviews of the literature occupy the highest position in currently proposed hierarchies of evidence. The aims of this study were to assess whether citation classics exist in published systematic review and meta-analysis (SRM), examine the characteristics of the most frequently cited SRM articles, and evaluate the contribution of different world regions.

**Methods:**

The 100 most cited SRM were identified in October 2012 using the Science Citation Index database of the Institute for Scientific Information. Data were extracted by one author. Spearman’s correlation was used to assess the association between years since publication, numbers of authors, article length, journal impact factor, and average citations per year.

**Results:**

Among the 100 citation classics, published between 1977 and 2008, the most cited article received 7308 citations and the least-cited 675 citations. The average citations per year ranged from 27.8 to 401.6. First authors from the USA produced the highest number of citation classics (n=46), followed by the UK (n=28) and Canada (n=15). The 100 articles were published in 42 journals led by the Journal of the American Medical Association (n=18), followed by the British Medical Journal (n=14) and The Lancet (n=13). There was a statistically significant positive correlation between number of authors (Spearman’s rho=0.320, p=0.001), journal impact factor (rho=0.240, p=0.016) and average citations per year. There was a statistically significant negative correlation between average citations per year and year since publication (rho = -0.636, p=0.0001). The most cited papers identified seminal contributions and originators of landmark methodological aspects of SRM and reflect major advances in the management of and predisposing factors for chronic diseases.

**Conclusions:**

Since the late 1970s, the USA, UK, and Canada have taken leadership in the production of citation classic papers. No first author from low or middle-income countries (LMIC) led one of the most cited 100 SRM.

## Background

Systematic reviews of the literature occupy the highest position in currently proposed hierarchies of evidence [1] and occupy this top position for two fundamental premises. Firstly, a systematic review involves the application of scientific strategies which limit bias by systematic assembly, critical appraisal and synthesis of relevant studies on a particular topic [2,3]. Secondly, reviews that include a meta-analysis provide precise estimates of the association studied[4]. Because of the importance of systematic reviews in summarizing the advances of health care knowledge, their number is growing rapidly [4]. If systematic reviews in fact represent the best level of evidence, they are likely to have great clinical importance[4]. It follows that they may be cited often in the literature. The acknowledgement that one article gives to another is a reference; the acknowledgement that one article receives from another is a citation [5]. The number of citations an article receives after publication reflects its impact on the scientific community. There have been a few recent attempts to identify and analyze “the most cited articles” in various specialties [5–9]. However, an analysis of the most frequently cited systematic review and meta-analysis (SRM) articles has not yet been reported. Montori and colleagues examined whether systematic reviews receive more citations than narrative reviews [4]. They found that rigorous systematic reviews were cited significantly more often than narrative reviews. In this paper we sought to identify and examine the characteristics of the most cited SRM related articles, such as ranking, year of publication, publishing journal, topic and contribution of different world regions to most cited SRM articles.

In addition, we assessed whether there was an association between year of publication, number of authors, number of pages, journals’ impact factor, and average citations per year.

## Methods

The Science Citation Index of the Institute for Scientific Information (ISI) was searched in October 2012 for systematic review and meta-analysis related articles. We searched for articles using validated keywords for identifying SRM [10]. To accredit an article to countries, the method of "absolute country counting" was adopted, in which each country contributing to an article received one paper credit based on the lead author’s correspondence or reprint address [11]. The 100 most-cited articles were selected for further descriptive analyses. Data collected included the year of publication, the topic covered, lead author’s correspondence country of origin, and number of citations. 

We used a density-equalizing map to visualize the citation classics by the corresponding address of the author. We used Gastner and Newman's algorithm [12] in order to produce a map of the world in which countries were re-sized according to the number of most cited SRM articles. These calculations employ a diffusion equation in the Fourier domain borrowed from elementary physics, which allows variable resolution by tracking moving boundaries [12].

The impact factors and immediacy factors of journals listed in the 2012 Journal Citation Reports Science Edition were adopted as quantitative tools for evaluating journals in which these articles were published. A journal’s impact factor is a measure of the frequency with which the "average article" in the journal has been cited in a given period of time. The impact factor for a journal is calculated based on a three-year period, and can be considered to be the average number of times published papers are cited up to two years after publication. Non-parametric (Spearman’s) correlation was used to assess the association between years since publication (with reference to the year 2012), numbers of authors, article’s length (number of pages), journal’s impact factor and average citations per year. 

## Results

**Figure 1:**
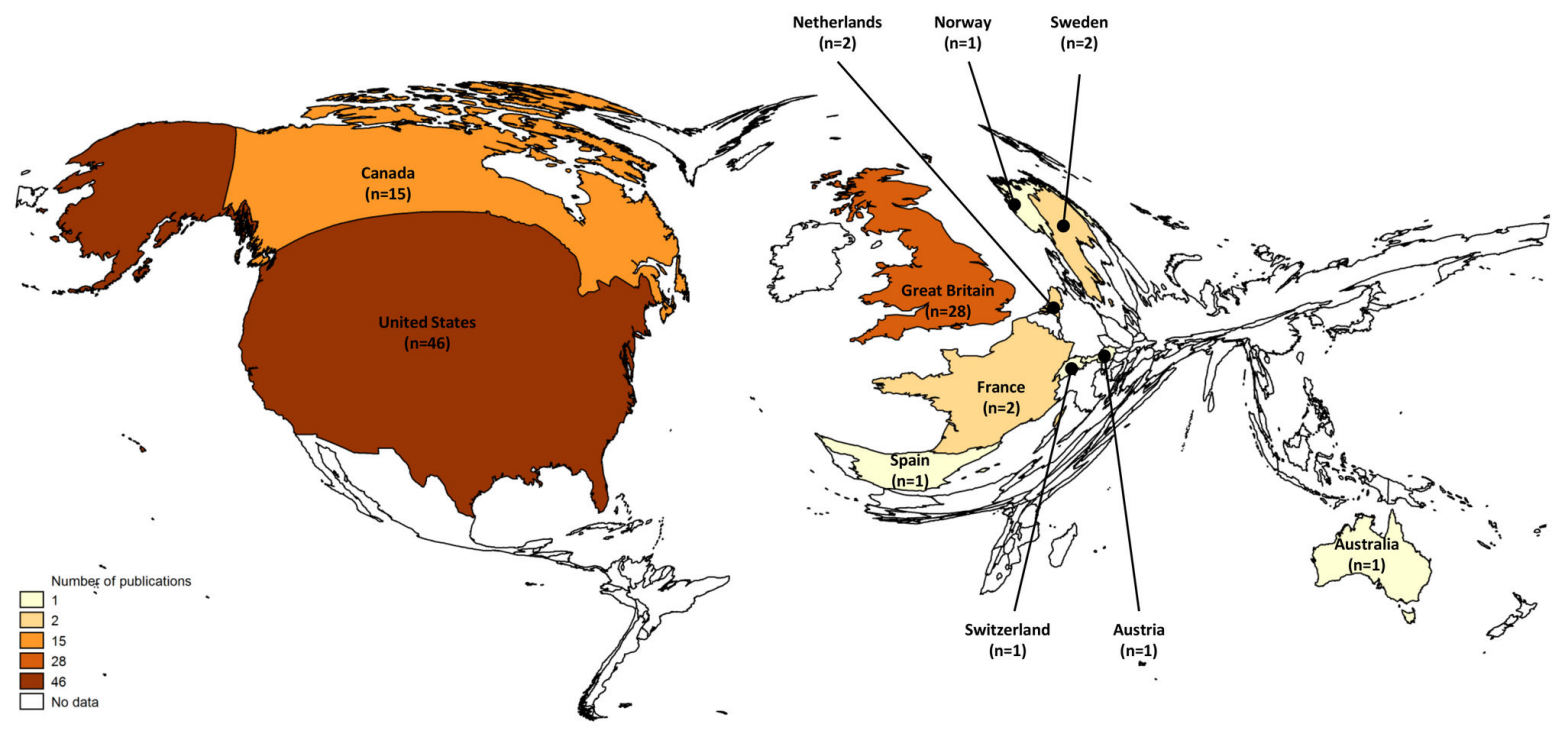
Spatial distribution of the 100 most frequently cited systematic reviews and meta-analyses related articles using density equalizing mapping. The area of each country were re-sized in proportion to its total number of 100 most frequently cited systematic reviews and meta-analyses related articles.

The 100 articles are listed in Table 1 in descending order, ranked according to the total number of citations since publication. Among the 100 citation classics, the most cited article received 7308 citations, and the least-cited 675 citations. The average citations per year ranged from 27.8 to 401.6. Figure 1 shows the density-equalizing map illustrating the number of contributions for each country in SRM citation classics. Density equalising mapping demonstrates that a relatively small number of countries were responsible for the majority of the top cited SRM articles (Figure 1). First authors from the USA produced the highest citation classics (n=46), followed by the UK (n=28) and Canada (n=15) (Figure 1). All the 100 most cited articles were published in the English Language.

**Table 1:** The 100 most frequently cited systematic review and meta-analysis articles.

**Articles**	**Year**	**Total no. of citations**		**Average citations per year**	
		**No**	**Rank**	**No**	**Rank**
Dersimonian R, Laird N: **Metaanalysis in Clinical-Trials**. *Controlled Clinical Trials* 1986, **7**: 177-188.	1986	7308	1	281.1	7
Egger M, Smith GD, Schneider M, Minder C: **Bias in meta-analysis detected by a simple, graphical test**. *British Medical Journal* 1997, **315**: 629-634.	1997	5197	2	346.5	2
Jadad AR, Moore RA, Carroll D, Jenkinson C, Reynolds DJM, Gavaghan DJ *et al*.: **Assessing the quality of reports of randomized clinical trials: Is blinding necessary**? *Controlled Clinical Trials* 1996, 17: 1-12.	1996	4535	3	283.4	6
Higgins JPT, Thompson SG, Deeks JJ, Altman DG: **Measuring inconsistency in meta-analyses**. *British Medical Journal* 2003, **327**: 557-560.	2003	4111	4	456.8	1
Schulz KF, Chalmers I, Hayes RJ, Altman DG: **Empirical-Evidence of Bias - Dimensions of Methodological Quality Associated with Estimates of Treatment Effects in Controlled Trials**. *Jama-Journal of the American Medical Association* 1995, **273**: 408-412.	1995	2884	5	169.6	17
Sackett DL, Rosenberg WMC, Gray JAM, Haynes RB, Richardson WS: **Evidence based medicine: What it is and what it isn't - It's about integrating individual clinical expertise and the best external evidence**. *British Medical Journal* 1996, **312**: 71-72.	1996	2881	6	180.1	14
Higgins JPT, Thompson SG: **Quantifying heterogeneity in a meta-analysis**. *Statistics in Medicine* 2002, 21: 1539-1558.	2002	2755	7	275.5	8
Baigent C, Keech A, Kearney PM, Blackwell L, Buck G, Pollicino C *et al.*: **Efficacy and safety of cholesterol-lowering treatment: prospective meta-analysis of data from 90,056 participants in 14 randomised trials of statins**. *Lancet* 2005, **366**: 1267-1278.	2002	2640	8	264	9
Boushey CJ, Beresford SAA, Omenn GS, Motulsky AG: **A Quantitative Assessment of Plasma Homocysteine As A Risk Factor for Vascular-Disease - Probable Benefits of Increasing Folic-Acid Intakes**. *Jama-Journal of the American Medical Association* 1995, **274**: 1049-1057.	1995	2532	9	148.9	19
Abe O, Abe R, Enomoto K, Kikuchi K, Koyama H, Masuda H *et al.*: **Effects of chemotherapy and hormonal therapy for early breast cancer on recurrence and 15-year survival: an overview of the randomised trials**. *Lancet* 2005, **365**: 1687-1717.	2005	2356	10	336.6	3
Moher D, Pham B, Jones A, Cook DJ, Jadad AR, Moher M *et al.*: **Does quality of reports of randomised trials affect estimates of intervention efficacy reported in meta-analyses**? *Lancet* 1998, **352**: 609-613.	1999	2335	11	179.6	15
Stroup DF, Berlin JA, Morton SC, Olkin I, Williamson GD, Rennie D *et al.*: **Meta-analysis of observational studies in epidemiology - A proposal for reporting**. *Jama-Journal of the American Medical Association* 2000, **283**: 2008-2012.	2000	2335	12	194.6	12
Lewington S, Clarke R, Qizilbash N, Peto R, Collins R: **Age-specific relevance of usual blood pressure to vascular mortality: a meta-analysis of individual data for one million adults in 61 prospective studies**. *Lancet* 2002, **360**: 1903-1913.	2002	2328	13	232.8	10
Alberti W, Anderson G, Bartolucci A, Bell D, Villalba JB, Brodin O *et al.*: **Chemotherapy in Non-Small-Cell Lung-Cancer - A Metaanalysis Using Updated Data on Individual Patients from 52 Randomized Clinical-Trials**. *British Medical Journal* 1995, **311**: 899-909.	1995	2087	14	122.8	24
Baigent C, Sudlow C, Collins R, Peto R: **Collaborative meta-analysis of randomised trials of antiplatelet therapy for prevention of death, myocardial infarction, and stroke in high risk patients**. *British Medical Journal* 2002, **324**: 71-86.	2005	2040	15	291.4	5
Block G, Patterson B, Subar A: **Fruit, Vegetables, and Cancer Prevention - A Review of the Epidemiologic Evidence**. *Nutrition and Cancer-an International Journal* 1992, 18: 1-29.	1992	1826	16	91.3	35
Begg CB, Mazumdar M: **Operating Characteristics of A Bank Correlation Test for Publication Bias**. *Biometrics* 1994, 50: 1088-1101.	1994	1756	17	97.6	31
Lazarou J, Pomeranz BH, Corey PN: **Incidence of adverse drug reactions in hospitalized patients - A meta-analysis of prospective studies**. *Jama-Journal of the American Medical Association* 1998, **279**: 1200-1205.	1998	1707	18	121.9	25
Buchwald H, Avidor Y, Braunwald E, Jensen MD, Pories W, Fahrbach K *et al.*: **Bariatric surgery: A systematic review and meta-analysis**. *Jama-Journal of the American Medical Association* 2004, **292**: 1724-1737.	2004	1706	19	213.3	11
Grady D, Rubin SM, Petitti DB, Fox CS, Black D, Ettinger B *et al.*: **Hormone-Therapy to Prevent Disease and Prolong Life in Postmenopausal Women**. *Annals of Internal Medicine* 1992, **117**: 1016-1037.	1992	1702	20	85.1	41
Marshall D, Johnell O, Wedel H: **Meta-analysis of how well measures of bone mineral density predict occurrence of osteoporotic fractures**. *British Medical Journal* 1996, **312**: 1254-1259.	1996	1583	21	98.9	30
Barrick MR, Mount MK: **The Big 5 Personality Dimensions and Job-Performance - A Metaanalysis**. *Personnel Psychology* 1991, 44: 1-26.	1991	1582	22	75.3	51
Davis DA, Thomson MA, Oxman AD, Haynes RB: **Changing Physician Performance - A Systematic Review of the Effect of Continuing Medical-Education Strategies**. *Jama-Journal of the American Medical Association* 1995, **274**: 700-705.	1995	1577	23	92.8	34
Appleby P, Baigent C, Collins R, Flather M, Parish S, Peto R *et al.*: **Indications for Fibrinolytic Therapy in Suspected Acute Myocardial-Infarction - Collaborative Overview of Early Mortality and Major Morbidity Results from All Randomized Trials of More Than 1000 Patients**. *Lancet* 1994, **343**: 311-322.	1994	1519	24	84.4	43
Nissen SE, Wolski K: **Effect of rosiglitazone on the risk of myocardial infarction and death from cardiovascular causes**. *New England Journal of Medicine* 2007, **356**: 2457-2471.	2007	1507	25	301.4	4
Grimshaw JM, Russell IT: **Effect of Clinical Guidelines on Medical-Practice - A Systematic Review of Rigorous Evaluations**. *Lancet* 1993, **342**: 1317-1322.	1993	1377	26	72.5	55
Abe O, Abe R, Enomoto K, Kikuchi K, Koyama H, Masuda H *et al.*: **Effects of radiotherapy and of differences in the extent of surgery for early breast cancer on local recurrence and 15-year survival: an overview of the randomised trials**. *Lancet* 2005, **366**: 2087-2106.	2005	1324	27	189.1	13
Danesh J, Whincup P, Walker M, Lennon L, Thomson A, Appleby P *et al.*: **Low grade inflammation and coronary heart disease: prospective study and updated meta-analyses**. *British Medical Journal* 2000, **321**: 199-204.	2004	1277	28	159.6	18
Farrer LA, Cupples LA, Haines JL, Hyman B, Kukull WA, Mayeux R *et al.*: **Effects of age, sex, and ethnicity on the association between apolipoprotein E genotype and Alzheimer disease - A meta-analysis**. *Jama-Journal of the American Medical Association* 1997, **278**: 1349-1356.	1997	1265	29	84.3	44
Moher D, Cook DJ, Eastwood S, Olkin I, Rennie D, Stroup DF: **Improving the quality of reports of meta-analyses of randomised controlled trials: the QUOROM statement**. *Lancet* 1999, **354**: 1896-1900.	1998	1262	30	90.1	37
Armitage CJ, Conner M: **Efficacy of the theory of planned behaviour: A meta-analytic review**. *British Journal of Social Psychology* 2001, 40: 471-499.	2001	1234	31	112.2	28
Dellinger RP, Carlet JM, Masur H, Gerlach H, Calandra T, Cohen J *et al.*: **Surviving Sepsis Campaign guidelines for management of severe sepsis and septic shock**. *Critical Care Medicine* 2004, 32: 858-873.	2004	1172	32	146.5	20
Pignon JP, Bourhis J, Domenge C, Designe L: **Chemotherapy added to locoregional treatment for head and neck squamous-cell carcinoma: three meta-analyses of updated individual data**. *Lancet* 2000, **355**: 949-955.	2000	1155	33	96.3	32
Guyatt G: **Evidence-Based Medicine - A New Approach to Teaching the Practice of Medicine**. *Jama-Journal of the American Medical Association* 1992, **268**: 2420-2425.	1992	1145	34	57.3	66
Phan KL, Wager T, Taylor SF, Liberzon I: **Functional neuroanatomy of emotion: A meta-analysis of emotion activation studies in PET and fMRI**. *Neuroimage* 2002, 16: 331-348.	2002	1136	35	113.6	27
Kramer MS: **Determinants of Low Birth-Weight - Methodological Assessment and Meta-Analysis**. *Bulletin of the World Health Organization* 1987, 65: 663-737.	2003	1130	36	125.6	23
Lohmueller KE, Pearce CL, Pike M, Lander ES, Hirschhorn JN: **Meta-analysis of genetic association studies supports a contribution of common variants to susceptibility to common disease**. *Nature Genetics* 2003, 33: 177-182.	1987	1130	37	45.2	81
Easterbrook PJ, Berlin JA, Gopalan R, Matthews DR: **Publication Bias in Clinical Research**. *Lancet* 1991, **337**: 867-872.	1991	1119	38	53.3	73
Anderson JW, Johnstone BM, Cooknewell ME: **Metaanalysis of the Effects of Soy Protein-Intake on Serum-Lipids**. *New England Journal of Medicine* 1995, **333**: 276-282.	1995	1113	39	65.5	60
Danesh J, Collins R, Appleby P, Peto R: **Association of fibrinogen, C-reactive protein, albumin, or leukocyte count with coronary heart disease - Meta-analyses of prospective studies**. *Jama-Journal of the American Medical Association* 1998, **279**: 1477-1482.	1998	1102	40	78.7	48
Juni P, Witschi A, Bloch R, Egger M: **The hazards of scoring the quality of clinical trials for meta-analysis**. *Jama-Journal of the American Medical Association* 1999, **282**: 1054-1060.	2001	1058	41	96.2	33
Atkins D, Best D, Briss PA, Eccles M, Falck-Ytter Y, Flottorp S *et al.*: **Grading quality of evidence and strength of recommendations**. *British Medical Journal* 2004, **328**: 1490-1494.	2004	1012	42	126.5	22
Smith ML, Glass GV: **Meta-Analysis of Psychotherapy Outcome Studies**. *American Psychologist* 1977, 32: 752-760.	1977	979	43	28	99
Brewin CR, Andrews B, Valentine JD: **Meta-analysis of risk factors for posttraumatic stress disorder in trauma-exposed adults**. *Journal of Consulting and Clinical Psychology* 2000, 68: 748-766.	2000	972	44	81	46
Bero LA, Grilli R, Grimshaw JM, Harvey E, Oxman AD, Thomson MA: **Getting research findings into practice - Closing the gap between research and practice: an overview of systematic reviews of interventions to promote the implementation of research findings**. *British Medical Journal* 1998, **317**: 465-468.	1998	964	45	68.9	57
Dickersin K, Scherer R, Lefebvre C: **Systematic Reviews - Identifying Relevant Studies for Systematic Reviews**. *British Medical Journal* 1994, **309**: 1286-1291.	1994	952	46	52.9	74
Anderson RJ, Freedland KE, Clouse RE, Lustman PJ: **The prevalence of comorbid depression in adults with diabetes - A meta-analysis**. *Diabetes Care* 2001, 24: 1069-1078.	2001	933	47	84.8	42
Oxman AD, Thomson MA, Davis DA, Haynes RB: **No Magic Bullets - A Systematic Review of 102 Trials of Interventions to Improve Professional Practice**. *Canadian Medical Association Journal* 1995, **153**: 1423-1431.	1995	928	48	54.6	72
Ohara MW, Swain AM: **Rates and risk of postpartum depression - A meta-analysis**. *International Review of Psychiatry* 1996, **8**: 37-54.	1996	913	49	57.1	67
Clarke R, Collins R, Lewington S, Donald A, Alfthan G, Tuomilehto J *et al.*: **Homocysteine and risk of ischemic heart disease and stroke - A meta-analysis**. *Jama-Journal of the American Medical Association* 2002, **288**: 2015-2022.	2002	910	50	91	36
DiMatteo MR, Lepper HS, Croghan TW: **Depression is a risk factor for noncompliance with medical treatment - Meta-analysis of the effects of anxiety and depression on patient adherence**. *Archives of Internal Medicine* 2000, **160**: 2101-2107.	2000	900	51	75	52
Colditz GA, Brewer TF, Berkey CS, Wilson ME, Burdick E, Fineberg HV *et al.*: **Efficacy of Bcg Vaccine in the Prevention of Tuberculosis - Metaanalysis of the Published Literature**. *Jama-Journal of the American Medical Association* 1994, **271**: 698-702.	1994	890	52	49.4	79
Wald DS, Law M, Morris JK: **Homocysteine and cardiovascular disease: evidence on causality from a meta-analysis**. *British Medical Journal* 2002, **325**: 1202-1206K.	2002	872	53	87.2	39
Danesh J, Wheeler JG, Hirschfield GM, Eda S, Eiriksdottir G, Rumley A *et al.*: **C-reactive protein and other circulating markers of inflammation in the prediction of coronary heart disease**. *New England Journal of Medicine* 2004, **350**: 1387-1397.	2000	860	54	71.7	56
Gabriel SE, Jaakkimainen L, Bombardier C: **Risk for Serious Gastrointestinal Complications Related to Use of Nonsteroidal Antiinflammatory Drugs - A Metaanalysis**. *Annals of Internal Medicine* 1991, **115**: 787-796.	1991	854	55	40.7	88
Harris EC, Barraclough B: **Suicide as an outcome for mental disorders - A meta-analysis**. *British Journal of Psychiatry* 1997, **170**: 205-228.	1997	850	56	56.7	69
Linn MC, Petersen AC: **Emergence and Characterization of Sex-Differences in Spatial Ability - A Meta-Analysis**. *Child Development* 1985, 56: 1479-1498.	1985	850	57	31.5	98
Bongartz T, Sutton AJ, Sweeting MJ, Buchan I, Matteson EL, Montori V: **Anti-TNF antibody therapy in rheumatoid arthritis and the risk of serious infections and malignancies - Systematic review and meta-analysis of rare harmful effects in randomized controlled trials**. *Jama-Journal of the American Medical Association* 2006, **295**: 2275-2285.	2006	846	58	141	21
Mcgeer PL, Schulzer M, Mcgeer EG: **Arthritis and anti-inflammatory agents as possible protective factors for Alzheimer's disease: A review of 17 epidemiologic studies**. *Neurology* 1996, 47: 425-432.	1996	826	59	51.6	77
Mensink RP, Katan MB: **Effect of Dietary Fatty-Acids on Serum-Lipids and Lipoproteins - A Metaanalysis of 27** Trials. Arteriosclerosis and Thrombosis 1992, 12: 911-919.	1992	822	60	41.1	87
Sheppard BH, Hartwick J, Warshaw PR: **The Theory of Reasoned Action - A Meta-Analysis of Past Research with Recommendations for Modifications and Future-Research**. *Journal of Consumer Research* 1988, 15: 325-343.	1988	822	61	34.3	95
Strong WB, Malina RM, Blimkie CJR, Daniels SR, Dishman RK, Gutin B *et al.*: **Evidence based physical activity for school-age youth**. *Journal of Pediatrics* 2005, **146**: 732-737.	2005	815	62	116.4	26
Colquitt JA, Conlon DE, Wesson MJ, Porter COLH, Ng KY: **Justice at the millennium: A meta-analytic review of 25 years of organizational justice research**. *Journal of Applied Psychology* 2001, 86: 425-445.	2001	811	63	73.7	54
Sacks HS, Berrier J, Reitman D, Anconaberk VA, Chalmers TC: **Meta-Analyses of Randomized Controlled Trials**. *New England Journal of Medicine* 1987, **316**: 450-455.	1987	810	64	32.4	97
Llovet JM, Bruix J: **Systematic review of randomized trials for unresectable hepatocellular carcinoma: Chemoembolization improves survival**. *Hepatology* 2003, 37: 429-442.	2003	804	65	89.3	38
Peyron R, Laurent B, Garcia-Larrea L: **Functional imaging of brain responses to pain. A review and meta-analysis (2000**). *Neurophysiologie Clinique-Clinical Neurophysiology* 2000, 30: 263-288.	2000	801	66	66.8	58
Lipsey MW, Wilson DB: **The Efficacy of Psychological, Educational, and Behavioral Treatment - Confirmation from Metaanalysis**. *American Psychologist* 1993, 48: 1181-1209.	1993	797	67	41.9	85
Dahlof B, Pennert K, Hansson L: **Reversal of Left-Ventricular Hypertrophy in Hypertensive Patients - A Metaanalysis of 109 Treatment Studies**. *American Journal of Hypertension* 1992, **5**: 95-110.	1992	796	68	39.8	89
Ernst E, Resch KL: **Fibrinogen As A Cardiovascular Risk Factor - A Metaanalysis and Review of the Literature**. *Annals of Internal Medicine* 1993, **118**: 956-963.	1993	794	69	41.8	86
Antman EM, Lau J, Kupelnick B, Mosteller F, Chalmers TC: **A Comparison of Results of Metaanalyses of Randomized Control Trials and Recommendations of Clinical Experts - Treatments for Myocardial-Infarction**. *Jama-Journal of the American Medical Association* 1992, **268**: 240-248.	1992	782	70	39.1	91
Patrick DL, Cheadle A, Thompson DC, Diehr P, Koepsell T, Kinne S: **The Validity of Self-Reported Smoking - A Review and Metaanalysis**. *American Journal of Public Health* 1994, 84: 1086-1093.	1994	777	71	43.2	83
Maron BJ: **Hypertrophic cardiomyopathy - A systematic review**. *Jama-Journal of the American Medical Association* 2002, **287**: 1308-1320.	2002	773	72	77.3	49
Miller ER, Pastor-Barriuso R, Dalal D, Riemersma RA, Appel LJ, Guallar E: **Meta-analysis: High-dosage vitamin E supplementation may increase all-cause mortality**. *Annals of Internal Medicine* 2005, **142**: 37-46.	2005	764	73	109.1	29
Wright IC, Rabe-Hesketh S, Woodruff PWR, David AS, Murray RM, Bullmore ET: **Meta-analysis of regional brain volumes in schizophrenia**. *American Journal of Psychiatry* 2000, **157**: 16-25.	2000	763	74	63.6	62
Horvath AO, Symonds BD: **Relation Between Working Alliance and Outcome in Psychotherapy - A Metaanalysis**. *Journal of Counseling Psychology* 1991, 38: 139-149.	1991	761	75	36.2	94
Voyer D, Voyer S, Bryden MP: **Magnitude of Sex-Differences in Spatial Abilities - A Metaanalysis and Consideration of Critical Variables**. *Psychological Bulletin* 1995, **117**: 250-270.	1995	756	76	44.5	82
Wong DKH, Cheung AM, Orourke K, Naylor CD, Detsky AS, Heathcote J: **Effect of Alpha-Interferon Treatment in Patients with Hepatitis-B E-Antigen-Positive Chronic Hepatitis-B - A Metaanalysis**. *Annals of Internal Medicine* 1993, **119**: 312-323.	1993	752	77	39.6	90
Capes SE, Hunt D, Malmberg K, Gerstein HC: **Stress hyperglycaemia and increased risk of death after myocardial infarction in patients with and without diabetes: a systematic overview**. *Lancet* 2000, **355**: 773-778.	2000	746	78	62.2	64
Moore FA, Feliciano DV, Andrassy RJ, Mcardle AH, Booth FVM, Morgensteinwagner TB *et al.*: **Early Enteral Feeding, Compared with Parenteral, Reduces Postoperative Septic Complications - the Results of A Metaanalysis**. *Annals of Surgery* 1992, **216**: 172-183.	1992	740	79	37	93
Juni P, Altman DG, Egger M: **Systematic reviews in health care - Assessing the quality of controlled clinical trials**. *British Medical Journal* 2001, **323**: 42-46.	1999	737	80	56.7	68
Lijmer JG, Mol BW, Heisterkamp S, Bonsel GJ, Prins MH, van der Meulen JHP *et al.*: **Empirical evidence of design-related bias in studies of diagnostic tests**. *Jama-Journal of the American Medical Association* 1999, **282**: 1061-1066.	1999	731	81	56.2	70
Ozer EJ, Best SR, Lipsey TL, Weiss DS: **Predictors of posttraumatic stress disorder and symptoms in adults: A meta-analysis**. *Psychological Bulletin* 2003, **129**: 52-73.	2003	730	82	81.1	45
Wellman HM, Cross D, Watson J: **Meta-analysis of theory-of-mind development: The truth about false belief**. *Child Development* 2001, 72: 655-684.	2001	728	83	66.2	59
Cosgrove SE, Sakoulas G, Perencevich EN, Schwaber MJ, Karchmer AW, Carmeli Y: **Comparison of mortality associated with methicillin-resistant and methicillin-susceptible *Staphylococcus aureus* bacteremia: A meta-analysis**. *Clinical Infectious Diseases* 2003, 36: 53-59.	2003	725	84	80.6	47
Hart RG, Benavente O, McBride R, Pearce LA: **Antithrombotic therapy to prevent stroke in patients with atrial fibrillation: A meta-analysis**. *Annals of Internal Medicine* 1999, **131**: 492-501	1999	717	85	55.2	71
Berlin JA, Colditz GA: **A Metaanalysis of Physical-Activity in the Prevention of Coronary Heart-Disease**. *American Journal of Epidemiology* 1990, **132**: 612-628.	1990	713	86	32.4	96
Eaden JA, Abrams KR, Mayberry JF: **The risk of colorectal cancer in ulcerative colitis: a meta-analysis**. *Gut* 2001, **48**: 526-535.	2001	711	87	64.6	61
Neal B, MacMahon S, Chapman N, Cutler J, Fagard R, Neal B *et al.*: **Effects of ACE inhibitors, calcium antagonists, and other blood-pressure-lowering drugs: results of prospectively designed overviews of randomised trials**. *Lancet* 2000, **356**: 1955-1964.	2000	709	88	59.1	65
Stuck AE, Siu AL, Wieland GD, Adams J, Rubenstein LZ: **Comprehensive Geriatric Assessment - A Metaanalysis of Controlled Trials**. *Lancet* 1993, **342**: 1032-1036.	1993	709	89	37.3	92
Zeggini E, Scott LJ, Saxena R, Voight BF, Marchini JL, Hu T *et al.*: **Meta-analysis of genome-wide association data and large-scale replication identifies additional susceptibility loci for type 2 diabetes**. *Nature Genetics* 2008, 40: 638-645.	2008	706	90	176.5	16
Brattstrom L, Landgren F, Israelsson B, Lindgren A, Hultberg B, Andersson A *et al.*: **Lowering blood homocysteine with folic acid based supplements: meta-analysis of randomised trials**. *British Medical Journal* 1998, **316**: 894-898.	1998	697	91	49.8	78
Labbe KA, Detsky AS, Orourke K: **Metaanalysis in Clinical Research**. *Annals of Internal Medicine* 1987, **107**: 224-233.	1987	694	92	27.8	100
Hites RA: **Polybrominated diphenyl ethers in the environment and in people: A meta-analysis of concentrations**. *Environmental Science & Technology* 2004, 38: 945-956.	2004	692	93	86.5	40
Claxton AJ, Cramer J, Pierce C: A **systematic review of the associations between dose regimens and medication compliance**. *Clinical Therapeutics* 2001, 23: 1296-1310.	2001	691	94	62.8	63
Lexchin J, Bero LA, Djulbegovic B, Clark O: **Pharmaceutical industry sponsorship and research outcome and quality: systematic review**. *British Medical Journal* 2003, **326**: 1167-1170B.	2003	689	95	76.6	50
Hunt DL, Haynes RB, Hanna SE, Smith K: **Effects of computer-based clinical decision support systems on physician performance and patient outcomes - A systematic review**. *Jama-Journal of the American Medical Association* 1998, **280**: 1339-1346.	1998	687	96	49.1	80
Deci EL, Koestner R, Ryan RM: **A meta-analytic review of experiments examining the effects of extrinsic rewards on intrinsic motivation**. *Psychological Bulletin* 1999, **125**: 627-668.	1999	685	97	52.7	75
Larosa JC, He J, Vupputuri S: **Effect of statins on risk of coronary disease - A meta-analysis of randomized controlled trials**. *Jama-Journal of the American Medical Association* 1999, **282**: 2340-2346.	1999	680	98	52.3	76
Kluger AN, DeNisi A: **The effects of feedback interventions on performance: A historical review, a meta-analysis, and a preliminary feedback intervention theory**. *Psychological Bulletin* 1996, **119**: 254-284.	1996	677	99	42.3	84
Lewis CM, Levinson DF, Wise LH, Delisi LE, Straub RE, Hovatta I *et al.*: **Genome scan meta-analysis of schizophrenia and bipolar disorder, part II: Schizophrenia**. *American Journal of Human Genetics* 2003, 73: 34-48.	2003	675	100	75	53

**Figure 2:**
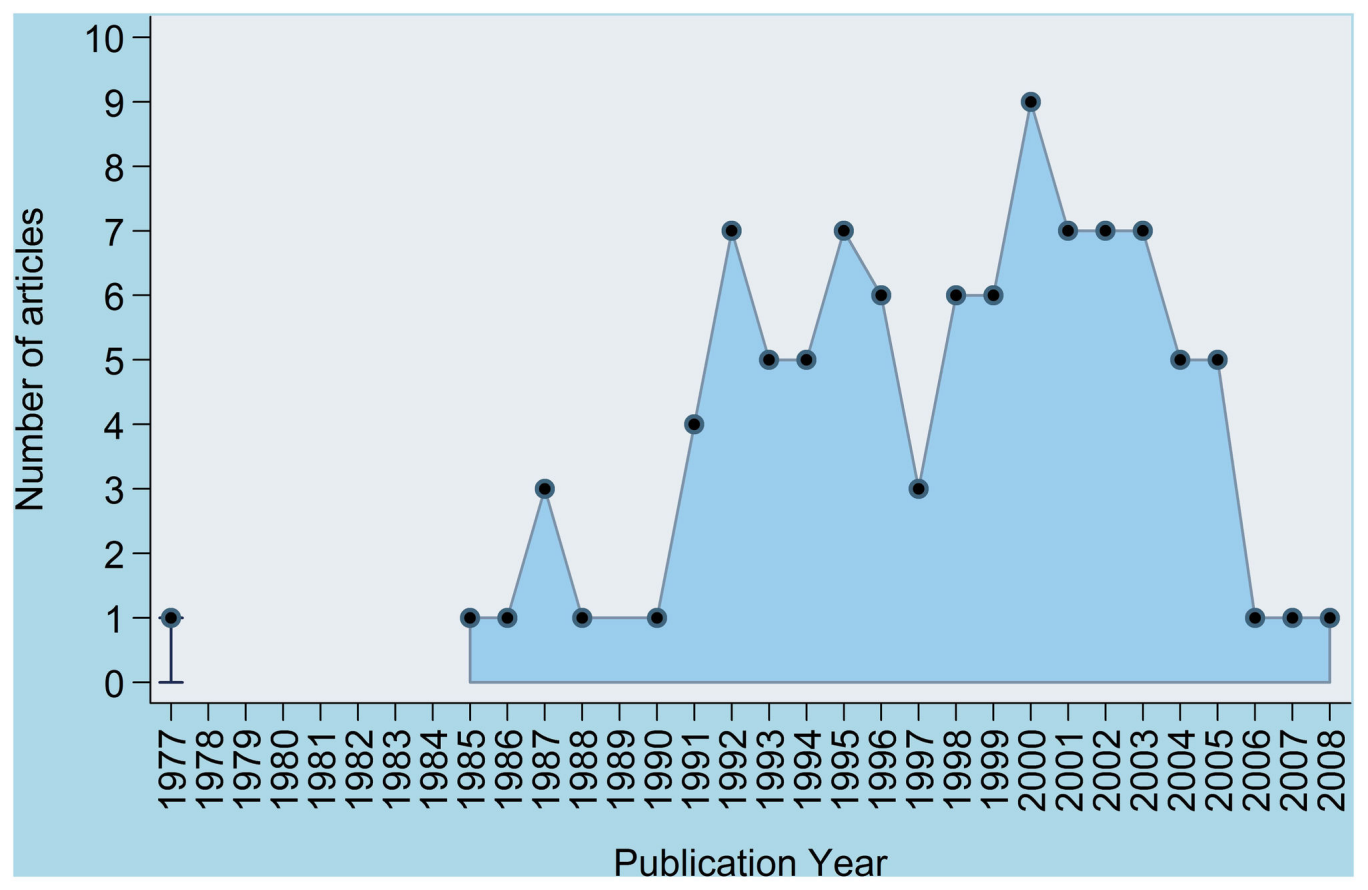
Graph demonstrating the time period of publication of the 100 most cited systematic reviews and meta-analyses related articles.

**Figure 3:**
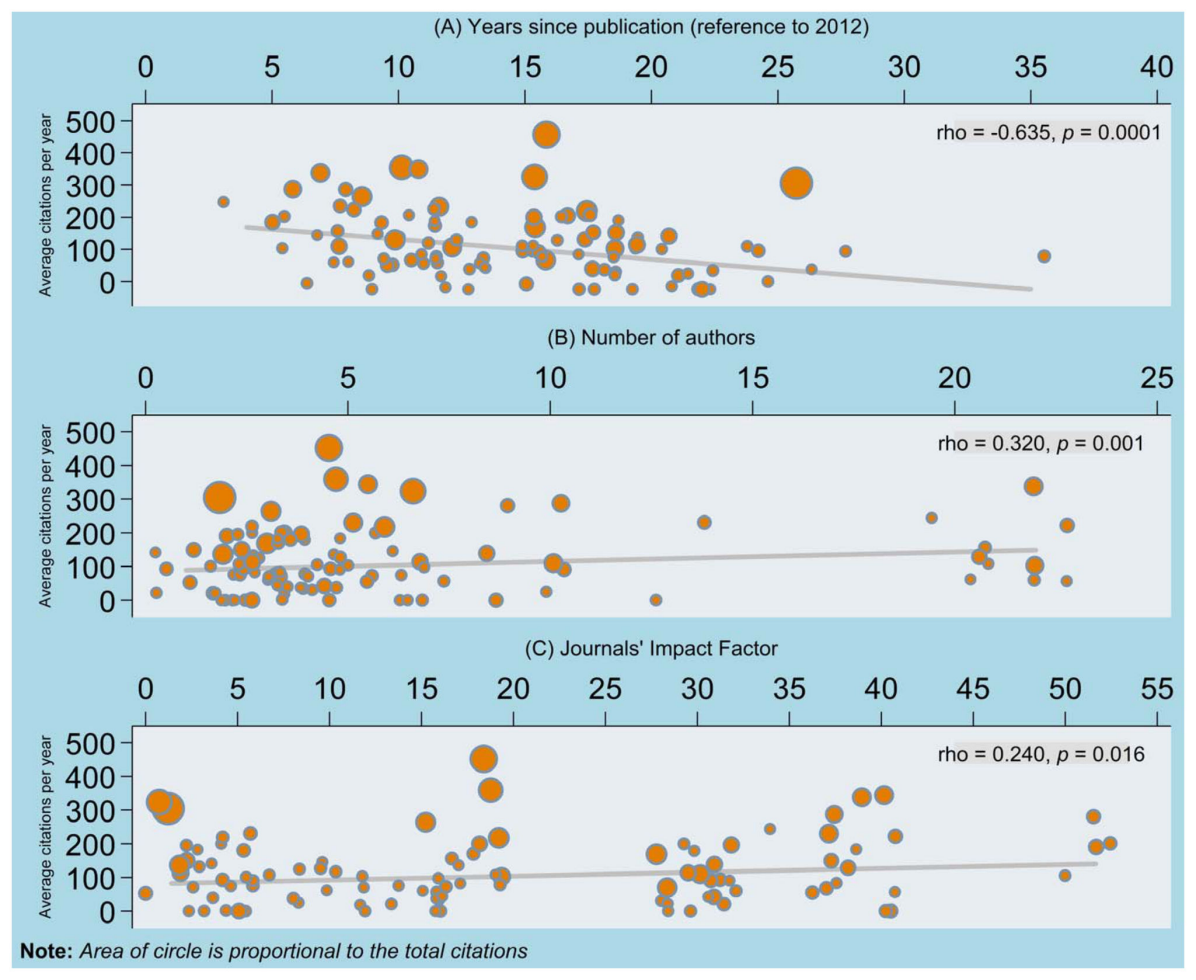
Correlation between since publication, number of authors, journals’ impact factor and average citation per year.

The year of publication with the relevant number of classics identified is shown in Figure 2. The oldest article was published in 1977 and the most recent article in 2008. Figure 3A shows correlation between average citations per year and year since publication (with reference to 2012). There was a statistically significant negative correlation between average citations per year and year since publication (Spearman’s rho = -0.636, 95% CI -0.739 to -0.501, p=0.0001), such that the average citations per year reduces with the number of years since publication.

The number of authors of the most cited articles ranged from one to 22. Four of the articles were authored by a single author and 18 articles by two authors. There was a statistically significant positive correlation between number of authors and average citations per year (rho=0.320, 95% CI 0.132 to 0.486, p=0.001), such that the greater the number of authors, the higher the average citation per year (Figure 3B). The median length of article was 10 pages (range: 2 to 75 pages). There was no statistically significant correlation between length and average citations per year (rho = -0.052, 95% CI -0.246 to 0.146, p=0.608). The most cited articles were published in 42 journals (Table 2), led by Journal of The American Medical Association (n=18) followed by the British Medical Journal (n=14), The Lancet (n=13), and the Annals of Internal Medicine (n=7). Journal impact factors ranged from 1.412 (for Biometrics) to 51.658 (for the New England Journal of Medicine). 

**Table 2:** Journals in which the most cited articles were published.

**Journal title**	**Number of articles**	**Impact factor (2012)**
Journal of The American Medical Association	18	29.978
British Medical Journal	14	17.215
Lancet	13	39.06
Annals of Internal Medicine	7	13.976
New England Journal of Medicine	4	51.658
Psychological Bulletin	4	15.575
American Psychologist	2	5.1
Child Development	2	4.915
Controlled Clinical Trials	2	1.597
Nature Genetics	2	35.209
American Journal of Epidemiology	1	4.78
American Journal of Human Genetics	1	11.202
American Journal of Hypertension	1	3.665
American Journal of Psychiatry	1	14.721
American Journal of Public Health	1	3.93
Annals of Surgery	1	6.329
Archives of Internal Medicine	1	11.462
Arteriosclerosis And Thrombosis	1	6.338
Biometrics	1	1.412
British Journal of Psychiatry	1	6.606
British Journal of Social Psychology	1	1.816
Bulletin of The World Health Organization	1	5.25
Canadian Medical Association Journal	1	6.465
Clinical Infectious Diseases	1	9.374
Clinical Therapeutics	1	2.23
Critical Care Medicine	1	6.124
Diabetes Care	1	7.735
Environmental Science Technology	1	5.257
Gut	1	10.732
Hepatology	1	12.003
International Review of Psychiatry	1	1.608
Journal of Applied Psychology	1	4.758
Journal of Consulting And Clinical Psychology	1	5.011
Journal of Consumer Research	1	3.542
Journal of Counseling Psychology	1	2.628
Journal of Pediatrics	1	4.035
Neuroimage	1	6.252
Neurology	1	8.249
Neurophysiologie Clinique Clinical Neurophysiology	1	2.553
Nutrition and Cancer An International Journal	1	2.695
Personnel Psychology	1	3.702
Statistics In Medicine	1	2.044

There was a statistically significant positive correlation between average citations per year and journal impact factor (rho=0. 240, 95% CI 0.045 to 0.416, p=0. 016) (Figure 3C). General and internal medicine were the main topics covered by these highly cited articles (n=59). Considerable attention was also given to Psychology and Psychiatry (n=13). 

The top-100 list contained landmark contributions dealing with methodological aspects of conducting systematic reviews and meta-analysis (n=17). At number 1, DerSimonian and Laird’s landmark article which introduced a novel simple random effects model for combining studies. Egger et al. (number-2) examined the prevalence of funnel plot asymmetry among published meta-analyses. Higgins et al. (number-4 and number-7) developed a new measure (*I* ^*2*^) for quantifying heterogeneity between studies included in a meta-analysis. Stroup et al. (number-12) reported a proposal for reporting meta-analyses of observational studies. The list of the most cited articles also reflects major advances in the management of non-communicable diseases (n=40) and in the identification of their predisposing factors for such diseases over the last 30 years. Baigent and colleagues (number-8) examined the efficacy and safety of statins on cholesterol lowering. Abe and co-researchers examined effects of chemotherapy and hormonal therapy for early breast cancer recurrence (number-10) and Lewington et al. (number-13) examined age-specific relevance of usual blood pressure and vascular mortality. 

## Discussion

This study identified and characterised the 100 most cited SRM related articles published in the past three decades, providing an overview of the citation frequency of these most cited articles. The list of the most cited articles identifies first authors and topics which reflect advances in methodological techniques in meta-analysis, major advances in the management of chronic diseases, and identification of predisposing factors over the last 30 years. Some of the most frequently cited articles were methodological papers. As expected the most highly cited papers were more likely to be published in journals high on the impact factor list [13,14]. It is important to note that, at present, no Cochrane review is among the 100 most cited SRM related articles. Cochrane reviews are systematic reviews of primary research in human health care and health policy, and are internationally recognised as the highest standard in evidence-based health care [15,16]. They investigate the effects of interventions for prevention, treatment and rehabilitation [15,16]. They also assess the accuracy of a diagnostic test for a given condition in a specific patient group and setting. They are published online in The Cochrane Library [15,16]. The low citations received by Cochrane reviews may be due to improper citations of Cochrane reviews, and the relatively recent tracking of Cochrane reviews by ISI. In addition, ISI Science Citation Index database covers all new and substantially updated Cochrane reviews from January 2005, and the first impact factor for Cochrane Database of Systematic Review was released in June 2008. 

We found that almost half of the most cited SRM related articles originated in the US. This Figure is comparable with the origin of citation classics in other fields [5–9]. The overwhelming influence the US has on medical research may be due to its large underlying population, enormous financial resources available to the scientific community in the country and its high population of active citing researchers compared to other countries [6,7]. Studies have demonstrated that biomedical research productivity worldwide is largely dependent on each country’s per capita gross national product and the expenditure allotted for research and development [17,18]. 

Our results support previous findings [5–9] that first authors from Africa, Asia, and South America had minimal or no contributions in the most cited articles. Scientific publishing activity worldwide over the past decades shows that most countries in these regions have low levels of publication [19]. The above finding is not a surprise because difficulties in research, publication, information access, and language barriers facing the least-developed countries are profound and seem almost intractable. Most information published in journals based in low and middle-income countries (LMICs) never leaves there home borders because these journals are largely excluded from major bibliographic databases. In addition, most of the reviews produced to date address health conditions that are priorities in the developed world[20]. Many major health concerns in LMICs have yet to be made the subject of a citation classic review, although there are signs that this may be changing[21]. 

In addition, the difficulties of conducting randomised controlled trials and other high quality studies in resource-poor situations result in the exclusion of many LMIC studies from systematic reviews [22]. However, there is a need to challenge the status quo. Scientists from these regions should forge multiple collaborations beyond historical, political, and cultural lines to share knowledge and expertise on SRM. In addition, there is a need to promote research in SRM in less developed regions of the world. This may involve but is not limited to the political will for research capacity development among LIMC health policymakers, the training of LMIC researchers to be competent in systematic review techniques, the development of infrastructure including research and academic institutes, the improvement of current collaborative partnerships with developed nations, increased sponsorship and support from world agencies such as the World Health Organization and the United Nations Organization.

Although we have tried to eliminate potential flaws in our citation analysis, some limitations were inevitable and are linked to the inherent problems of citation analysis [23,24]. The citation of a scientific article usually follows a time course, it is usually not cited until one to two years after publication, reaches a maximum after three to ten years, then declines [6]. Another problem is oriented or biased citing, including various types of conscious or unconscious biases, such as self-citation (bias towards one’s own work), in-house (bias towards friends or colleagues), journal or powerful person (bias towards reviewers, editors, members of grant awarding bodies), negative citation (bias towards potential negative credits), English language (bias towards publishing and referencing English articles), and omission bias (bias towards not referencing competitors or sources contradictory to one’s own results) [25]. Other limitations include the incorrect citation of origin for the authors. By using the author addresses listed in the bylines of research articles, one can only identify countries and organizations where the authors were employed when the research was done or where the article was written [18].

## Conclusion

Since the late 1970s, the USA, UK, and Canada have taken leadership in the production of citation classic papers. No author from LMICs led any of the most cited 100 SRM. There is a need to challenge the status quo. Scientists from LMIC should forge multiple collaborations to share knowledge and expertise on SRM. In addition, there is a need to strengthen research capacity in these countries and more support should be provided for the advancement of research efforts.
